# Microglial Ion Channels as Potential Targets for Neuroprotection in Parkinson's Disease

**DOI:** 10.1155/2013/587418

**Published:** 2013-10-30

**Authors:** Jason R. Richardson, Muhammad M. Hossain

**Affiliations:** Department of Environmental and Occupational Medicine, Rutgers-Robert Wood Johnson Medical School, Environmental and Occupational Health Sciences Institute, Rutgers, The State University of New Jersey, 170 Frelinghuysen Road, Piscataway, NJ 08854, USA

## Abstract

Parkinson's disease (PD) is a chronic, degenerative neurological disorder that is estimated to affect at least 1 million individuals in the USA and over 10 million worldwide. It is thought that the loss of neurons and development of inclusion bodies occur gradually over decades until they progress to the point where ~60% of the dopamine neurons are lost and patients present with motor dysfunction. At present, it is not clear what causes this progression, and there are no current therapies that have been successful in preventing PD progression. Although there are many hypotheses regarding the mechanism of PD progression, neuroinflammation may be a major contributor to PD pathogenesis. Indeed, activated microglia and subsequent neuroinflammation have been consistently associated with the pathogenesis of PD. Thus, interference with this process could provide a means of neuroprotection in PD. This review will discuss the potential of targeting microglia to reduce neuroinflammation in PD. Further, we discuss the potential of microglial ion channels to serve as novel targets for neuroprotection in PD.

## 1. Introduction

Parkinson's disease (PD) is a disabling neurodegenerative disorder, estimated to affect over 10 million people worldwide and over 1 million people in the United States. With the number of Americans over 65 rapidly increasing, it is inevitable that there will be a drastic rise in PD cases over the next 20 years [1]. PD presents clinically as bradykinesia, muscular rigidity, a resting tremor, and postural instability, all of which are the direct result of degeneration of dopaminergic neurons in the substantia nigra pars compacta (SNc). Neuropathologically, PD is characterized by the loss of pigmented neurons in the SNc, the presence of Lewy bodies, and cytoplasmic inclusions containing ubiquitin and *α*-synuclein [2]. Dopaminergic cell bodies in the SNc provide dopaminergic innervation to the striatum, and degeneration of these neurons results in dopamine depletion in the striatum. In turn, dopamine depletion and the loss of dopamine neurons lead to the hallmark motor dysfunctions of PD, typically after a loss of ~80% of striatal dopamine. Unfortunately, there are limited treatment options currently available for PD, and these treat the symptoms not the disease itself. Therefore, there is a significant need to find therapeutics that target the disease process itself. 

## 2. Neuroinflammation and Microglia in PD

**Figure 1:**
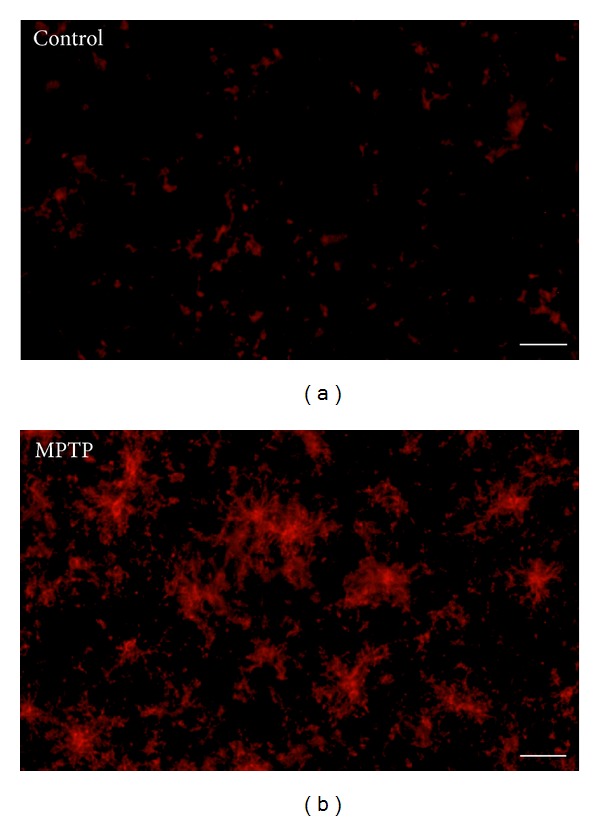
Acute MPTP-induced microglial activation in the striatum of adult mice. (a) Microglia in resting condition in control and (b) activated microglia in MPTP treated animals. MPTP was dissolved in physiological saline and administered subcutaneously (s.c.) a dose of 10 mg/kg every 2 hr for a total of 4 injections. Mice were killed 48 h after last injection and processed for immunofluorescence staining. Microglia were labeled with MAC-1 antibody. Scale bar = 400 *μ*m.

Although the precise mechanism(s) for neurodegeneration in PD is unknown, there is extensive evidence to suggest that neuroinflammation contributes to the pathogenic process of PD. The midbrain, which encompasses the SNc, contains a higher proportion of microglia, the resident immune cells of the brain, than other brain regions [3]. Postmortem PD brains display evidence of inflammation and oxidative stress, including increased microglial activation and lipid peroxidation [4, 5]. The landmark study by McGeer and coworkers [6] first described increased number of microglia in the substantia nigra of post-mortem PD patients. In humans, persistent neuroinflammation and sustained microglial activation were observed in post-mortem brains of humans who developed a Parkinsonian syndrome after accidentally injecting the neurotoxicant MPTP many years earlier [7]. Microglial activation also appears to be a contributing factor to dopaminergic neurodegeneration in animal models of PD, including those employing rotenone, MPTP (Figure 1), and paraquat [8–10]. Further, long-term increases in microglial activation following MPTP exposure were observed in non-human primates [11]. The finding of sustained microglial activation in postmortem samples and animal model has since been confirmed in living PD patients undergoing PET scans with the ligand PK11195 [12]. Thus, reducing or preventing sustained microglial activation may lead to reduction of neurodegeneration.

## 3. Clinical Trials Targeting Neuroinflammation in PD 

Early studies demonstrating elevated oxidative damage in PD led to the idea that antioxidants might be effective neuroprotective agents in PD. The most notable test of this hypothesis was the DATATOP trial, which tested the ability of vitamin E (2000 IU per day) to delay disease progression. Unfortunately, vitamin E was ineffective, and the study was stopped because of hepatotoxicity [13]. Several epidemiological studies reported that regular use of nonsteroidal anti-inflammatory drugs, particularly Ibuprofen, is associated with a lower risk of PD [14–16]. These findings led to renewed hope that targeting neuroinflammation would lead to neuroprotection in PD. 

There are currently several preclinical and clinical studies ongoing for neuroprotection in PD [17], including a prominent one based on the ability of the tetracycline antibiotic minocycline to reduce microglial activation. Early studies reported that minocycline reduced dopaminergic neurodegeneration in rodent models of PD through a reduction of microglial activation [18]. However, subsequent studies reported that minocycline exacerbated MPTP toxicity in both mice [19] and monkeys [20]. Yet another study reported that minocycline could indeed reduce microglial activation, based on morphological criteria, but did not prevent dopaminergic neurodegeneration following MPTP exposure, which was attributed to an inability to decrease release of TNF*α* [21]. There is also concern because a previous clinical trial for minocycline in amyotrophic lateral sclerosis had to be stopped because of disease acceleration [22] and that minocycline was ineffective in reducing clinical symptoms of multiple-system atrophy [23]. However, the ongoing clinical trial for minocycline in PD has yet to report results. 

## 4. Targeting the Consequences of Activated Microglia

Microglia, often referred to as the resident macrophages of the brain, play a key role in dopaminergic neurodegeneration [24]. Microglia can be activated by a number of signals, including lipopolysaccharide, which interacts with the Toll-like receptor, and can contribute to dopamine neuron death *in vitro* and *in vivo* [25]. Likewise, damaged neurons also release factors, such as *α*-synuclein, neuromelanin, and calpain, which activate microglia [26, 27]. This activation is characterized by an increase in number, changes in morphology to an irregular and elongated body and short processes, and intense labeling with Iba-1 (see Figure 1). During ongoing neuroinflammation, activated microglia produce a variety of proinflammatory mediators including reactive oxygen species (ROS) and nitric oxide (NO), along with a variety of cytokines, including TNF*α*. This in turn can lead to dopamine neuron death. Thus, there exists a vicious cycle between microglial activation and dopaminergic neurodegeneration that may contribute to the pathophysiology and progression of PD. 

For years, researchers used genetic or pharmacological means to target the untoward effects of microglial activation. Many of these studies reported neuroprotection in animal models of PD, but few have reached the point of clinical trials, and none have proven successful in the clinic to date. Here, we briefly review three of the most studied targets for neuroprotection in PD through reduction of neuroinflammation.

### 4.1. Nitric Oxide Production

Under pathological conditions, such as PD, nitric oxide (NO) produced by inducible nitric oxide synthases (iNOS) combines with superoxide to form the highly toxic peroxynitrite, which directly contribute to oxidative damage and neuroinflammation. In PD patients, there is increased immunoreactivity for iNOS and 3-nitrotyrosine in the substantia nigra, likely the result of microglial activation [28]. Increased iNOS and 3-nitrotyrosine are also found in ventral midbrain and striatum of MPTP-treated animals [29]. From a therapeutic standpoint, pretreatment of animals with iNOS inhibitors, such as 7-nitroindazole, or genetic deletion of iNOS was partially protective against MPTP and paraquat neurotoxicity [29]. However, iNOS inhibitors have not advanced into clinical trials for PD, mainly because of the potential for cardiotoxicity.

### 4.2. TNF*α* Production

Activated microglia release a number of cytokines and chemokines, most notably the pro-inflammatory cytokine TNF*α*. Studies in post-mortem and living PD patients consistently found that TNF*α* levels are elevated in the brain, serum, and cerebrospinal fluid [30]. In preclinical models, genetic deletion of TNF*α* or its receptors was partially protective against MPTP toxicity [31]. However, the use of anti-TNF therapeutics is hindered by poor penetration of the blood-brain barrier. Furthermore, recent reports of microglial heterogeneity and a potential role of TNF in cell survival have brought into question whether targeting TNF may actually be detrimental [32].

### 4.3. NADPH Oxidase Activation

NADPH oxidase, also known as NOX2, is a prime generator of ROS in microglia [33, 34]. NOX2 consists of multiple subunits, including gp91^phox^, which serves as the primary catalytic subunit [35–38]. NOX2 is expressed in a variety of cell types in the brain but has particularly high expression in microglia [39]. Microglial NOX2 is increased in post-mortem PD brains, as evidenced by increased immunostaining for gp91^phox^ [40]. The NADPH oxidase pathway influences dopaminergic neurodegeneration by both LPS and MPTP, as mice lacking NOX2 or the catalytic subunit gp91^phox^ exhibit reduced microglial activation and neurodegeneration [33, 41]. Subsequent studies reported that several nonspecific and relatively specific inhibitors of NOX2 were protective in preclinical models of PD, including dextromethorphan, the aforementioned minocycline, apocynin, and diphenyleneiodonium [42]. However, limitations in blood-brain-barrier permeability, potential off-target effects, lack of specificity, and potential disruption of the beneficial effects of NOX2 in the immune response have hampered the clinical development of NOX2 inhibitors.

## 5. Microglial Ion Channels as Potential New Targets to Reduce Neuroinflammation

Microglia express several ion channels, including K^+^, Ca^2+^, and Na^+^ channels, among others, that are increasingly being recognized for their potential to modulate microglial functions [43–48]. Early studies on membrane properties of microglial cells in culture demonstrated a preponderance of inward rectifying K^+^ currents and a resting membrane potential of approximately −50 mV [49]. Additional studies demonstrated that microglia isolated from neurosurgical samples in adults expressed Na^+^ currents. Here, we briefly discuss microglial K^+^, Ca^2+^, and Na^+^ channels and explore their potential as novel targets for neuroprotection.

### 5.1. Potassium Channels

K^+^ channels (K_v_), and in particular the inward rectifier K_v_ (K_IR_), were one of the first ion channels characterized in microglia [49]. Indeed, K_IR_ appear to be an early marker of activated microglia, as they are reported not to be expressed in resting microglia. There is also a delayed rectifying outward K^+^ current that is associated with activated microglia and appears to be mediated by K_v_ 1.3 and 1.5. K_v_ 1.3 was reported to be increased in LPS-activated microglia, as well as in microglia activated by HIV TAT and *β*-amyloid [46, 50]. LPS or phorbol ester-induced respiratory burst was blocked by a variety of K_v_ blockers, but these had no effect on NO production [51]. Given that these blockers are toxin based and K_v_ are also present on neurons, further research is needed to determine the potential of K_v_ as potential targets for neuroprotection *in vivo*.

The vast majority of recent focus on K_v_ in microglia has focused on the calcium-activated K^+^ channels, particularly KCNN4/KCa2 and 3.1, and ATP-sensitive K^+^ channels (K_ATP_) [52]. KCa3.1 was reported to contribute to microglia activation and NO-dependent neurodegeneration in retinal ganglion cells subjected to optic nerve transaction [53]. Importantly, neurodegeneration was reduced by intraocular injection of triarylmethane-34. With regards to K_ATP_ channels, there is more of a controversy over their effects. Most studies found that administration of diazoxide, a classic K_ATP_ channel activator, reduces microglial activation and is neuroprotective in a variety of models involving neuroinflammation [54]. However, a recent report found that blockade of the K_ATP_ channel with glibenclamide following hypoxia-ischemia is neuroprotective [55]. Given the nonspecific nature of the agonists and antagonists used and the presence of these K_ATP_ channels on neurons, further research is warranted on targeting these channels for neuroprotection. 

### 5.2. Calcium Channels

At this time, there is limited electrophysiological evidence for voltage-gated Ca^2+^ channels in microglia [56, 57]. However, treatment with the BAY K 8644, a positive modulator of voltage-gated Ca^2+^ channels, enhanced superoxide production in microglial cells that was blocked by nifedipine [56]. Calcium channel blockers, particularly of the L-type, have recently received significant attention as potential targets for neuroprotection in PD [58]. Indeed, administration of L-type Ca^2+^ channel antagonists, including isradipine [59] and nimodipine [60], exerts neuroprotective effects in MPTP mouse models. However, it is not clear whether this effect results from inhibition of Ca^2+^ channels on neurons or microglia.

There is also a intracellular Ca^2+^ release-activated Ca^2+^ current in microglia that appears to be regulated by Ora1 and TRP channels, particularly TRPM7 [61]. A growing body of evidence suggests that TRP channels regulate microglial function and may contribute to neurodegeneration [62]. As such, TRP channels may represent a new target for reducing neuroinflammation and exerting neuroprotective effects. 

### 5.3. Sodium Channels

Sodium channels (Na_v_) are ubiquitously expressed in neurons throughout the central and peripheral nervous systems where their primary function is to generate action potentials for cellular communication. However, Na_v_ are also expressed in other neuronal cells, such as astrocytes and microglia, where their role is still being established [63]. Recently, microglial ion channels, including Na_v_, were reported to participate in the regulation of a wide range of cellular functions in microglia, including morphological transformation, proliferation, migration, and phagocytosis in response to inflammatory stimuli [43, 45]. Additional studies demonstrated that a variety of Na_v_ blockers, including tetrodotoxin, and a variety of antiepileptic drugs reduce the phagocytic and migratory activity of cultured microglia [43]. Most recently, we demonstrated that increased tetrodotoxin-sensitive Na^+^ flux is an early response to LPS application in microglia and that tetrodotoxin can block TNF*α* secretion [64].

Using isoform-specific antibodies, Black and coworkers [43] reported that cultured rat microglia express Na_v_ 1.1, 1.5, and 1.6, with 1.6 being the most highly expressed isoform. Na_v_ 1.6 was also confirmed to be the isoform responsible for alteration of microglial function, as primary cultures from mice lacking Na_v_ 1.6 exhibited a reduction in LPS-stimulated phagocytosis [65]. *In vivo*, elevated expression of the Na_v_ 1.6 isoform was found in activated microglia in an animal model of experimental autoimmune encephalopathy (EAE) and in human multiple sclerosis lesions [65]. Indeed, this study found minimal to no staining of quiescent microglia with Na_v_. More importantly, this elevated expression was progressive, and Na_v_ blockers used clinically as antiepileptic drugs, including phenytoin, reduced microglial activation and axonal degeneration in this model. However, further studies found that if phenytoin treatment was removed, there was a rapid exacerbation of EAE symptoms that was accompanied by increased activated microglia [66]. The mechanism of this exacerbation remains to be fully established. 

## 6. Other Potential Targets on Microglia That Regulate Ionic Homeostasis

### 6.1. NHE and Na^**+**^/Ca^**2+**^ Exchangers

NHE are important regulators of intracellular pH through controlling transport of H^+^ against an influx of Na^+^ ions [67, 68]. In the brain, NHE-1 is the most abundant NHE isoform and regulates cytosolic pH in neurons, astrocytes, and microglia. Early studies demonstrated that increased NHE-1 activity during ischemia reperfusion in the heart and brain contributes to reversal of the Na^+^/Ca^2+^ exchanger and influx of Ca^2+^ leading to cell death [69, 70]. More recently, activation of microglia by LPS increases NADPH oxidase activity in microglia that is partially inhibited by NHE-1 inhibition [64, 68]. Luo and co-workers also reported that pharmacological inhibition of NHE-1 was partially neuroprotective against ischemic brain injury, in part through dampening the microglial response [41, 71, 72]. A similar effect was observed in mice heterozygous for NHE-1 [71]. Taken together, these data suggest that NHE-1 may be a viable target for neurodegeneration in PD. However, the clinical development of NHE-1 antagonists has been hampered by poor efficacy and significant side effects [73]. Likewise, a recent report demonstrated a neuroprotective effect of SEA0400, a Na^+^/Ca^2+^ exchanger inhibitor, in an MPTP model of PD [74]. While encouraging, this neuroprotective effect was not associated with decreased microglial activation, suggesting that it targets the neuronal Na^+^/Ca^2+^ exchanger.

### 6.2. Hv1 Proton Channels

An exciting new ion channel recently described in microglia is the Hv1 proton channel. Hv1 was first shown to be expressed in immune tissues and support the respiratory burst in phagocytic leukocytes [75]. Subsequent studies revealed the requirement of Hv1 for NADPH-oxidase generation of superoxide during the respiratory burst and a role for regulation of intracellular pH [76]. Most recently, Hv1 was reported to be selectively expressed in isolated human and mouse brain microglia [77]. Further, mice lacking Hv1 displayed less neurodegeneration following *in vitro* oxygen-glucose deprivation and *in vivo* following partial cerebral artery occlusion. These neuroprotective effects were associated with decreased NADPH-oxidase-dependent ROS production. Because Hv1 appears to be present only in brain microglia and is required for NADPH oxidase activation, it may be an ideal target for reducing microglial activation and subsequent neurodegeneration without the potential of off-target effects. However, this remains to be established since there may be infiltrating macrophages from the periphery that express Hv1.

## 7. Conclusions

A large and growing body of evidence supports an integral role for microglial activation and neuroinflammation in the pathogenesis of PD. Unfortunately, this information has not led to successful translation to clinical trials for neuroprotection in PD. There are numerous reasons for this lack of success in translation to the clinic, including pharmacokinetic issues. However, many of the bottlenecks arise from the fact that many of the targets are widely expressed, leading to adverse effects that preclude their use in PD. Emerging data on the presence of unique localization of ion channels on microglia and the potential for their expression to be increased in neurodegeneration may provide a new avenue for specifically targeting microglia and dampening the ongoing inflammatory process in PD. However, further work is required to determine whether ion channel expression or function in microglia is altered in PD and in which type of microglia (Th1 or Th2) they are expressed. In turn, this may provide additional means of targeting activated pro-inflammatory Th1 microglia and preserving the potential beneficial function of Th2 type.
